# Relationship between Uveal Inflammation and Viral Detection in 30 Cats with Feline Infectious Peritonitis

**DOI:** 10.3390/pathogens11080883

**Published:** 2022-08-05

**Authors:** Mariano Carossino, Fabio Del Piero, Jeongha Lee, David B. Needle, Jonathan M. Levine, Ronald R. Riis, Roger Maes, Annabel G. Wise, Keenan Mullaney, Jacqueline Ferracone, Ingeborg M. Langohr

**Affiliations:** 1 Department of Pathobiological Sciences and Louisiana Animal Disease Diagnostic Laboratory, Louisiana State University School of Veterinary Medicine, Baton Rouge, LA 70803, USA; 2 New Hampshire Veterinary Diagnostic Laboratory, College of Life Sciences and Agriculture, University of New Hampshire, Durham, NH 03824, USA; 3 Department of Small Animal Clinical Sciences, School of Veterinary Medicine and Biomedical Sciences, Texas A&M University, College Station, TX 77845, USA; 4 Department of Clinical Sciences, College of Veterinary Medicine, Cornell University, Ithaca, NY 14853, USA; 5 Veterinary Diagnostic Laboratory, College of Veterinary Medicine, Michigan State University, East Lansing, MI 48824, USA; 6 Department of Pathobiology and Diagnostic Investigation, College of Veterinary Medicine, Michigan State University, East Lansing, MI 48824, USA; 7 Washtenaw Technical Middle College, Ann Arbor, MI 48105, USA; 8 PennVet New Bolton Center, School of Veterinary Medicine, University of Pennsylvania, Kennett Square, PA 19348, USA

**Keywords:** feline infectious peritonitis (FIP), coronavirus, alphacoronavirus, uveitis, cat, immunohistochemistry, in situ hybridization, viral antigen, viral RNA

## Abstract

Feline infectious peritonitis (FIP) virus is the most common infectious cause of uveitis in cats. Confirmatory diagnosis is usually only reached at postmortem examination. The relationship between the histologic inflammatory pattern, which depends on the stage of the disease, and the likelihood of detection of the viral antigen and/or RNA has not been investigated. We hypothesized that viral detection rate by either immunohistochemistry, in situ hybridization or RT-qPCR is dependent upon the predominant type of uveal inflammatory response (i.e., pyogranulomatous vs. plasmacytic). Thus, the aims of this study were to evaluate cases of FIP-induced uveitis, localize the viral antigen and RNA, and assess the relationship between the inflammatory pattern (macrophage- vs. plasma cell-rich) and the likelihood of detecting the FIP antigen and/or RNA. We evaluated 30 cats with FIP-induced uveitis. The viral antigen and/or RNA were detected within uveal macrophages in 11/30 cases, of which 8 tested positive by RT-qPCR. Correlation analysis determined a weak to moderate but significant negative correlation between the degree of plasmacytic uveal inflammation and the likelihood of detecting the FIP antigen and RNA. This study suggests that predominance of plasmacytic inflammation in cases of FIP uveitis reduces the odds of a confirmatory diagnosis through the viral detection methods available.

## 1. Introduction

Feline infectious peritonitis (FIP) is a progressive and fatal systemic viral disease of domestic and wild felids that most commonly affects young cats. This specific disease entity develops following mutations in the genome of feline coronavirus (FCoV), a common and relatively apathogenic enteric virus in the genus *Alphacoronavirus* (subfamily *Orthocoronavirinae*, family *Coronaviridae*). Mutations leading to the emergence of FIP-associated coronavirus (FIPV) mostly occur de novo within each individual FCoV-infected cat, increasing virulence and triggering a systemic infection. FIPV has a complex pathogenesis primarily driven by a change in cellular tropism that allows the virus to infect and replicate within macrophages, spread systemically and induce an immune-mediated disease in affected cats responsible for variable lesions and clinical signs. It has been estimated that mutations of FCoV leading to FIP occur in roughly 10% of infected cats [1,2,3,4,5,6,7,8,9,10].

The monocyte/macrophage-tropic FIPV typically manifests as either “dry,” “wet” or as a mixed form. The “dry” or non-effusive form occurs with a weak cell-mediated immune response, inducing disseminated granulomatous perivascular inflammation (type IV hypersensitivity). The “wet” or effusive form, which is associated with an ineffective cell-mediated immune response and a strong antibody response, leads to the development of an immune complex disease (type III hypersensitivity) with vascular damage inducing severe effusions and fibrinous exudation [1,4,11]. The dry form is classically associated with meningitis, encephalitis and uveitis, and generally has a relatively longer survival rate than the wet form [1,4,11]. FIP is one of the most common causes of uveitis in cats [12,13,14,15,16,17]. A recent study detailed the characterization of the ocular lesions in FIPV-infected cats with uveitis [18]. That study determined the occurrence of retinal gliosis in affected cats and the predominance of B lymphocytes and plasma cells over macrophages within the uveal tract.

Antemortem diagnosis of FIP is often challenging, and there is currently no single, fully accurate diagnostic test [19,20,21,22,23]. While several reverse transcriptase real-time polymerase chain reaction (RT-qPCR) assays for the diagnosis of FIP are available, their clinical performance is variable, with false negative and false positive test results commonly encountered [19,20,21,22,23]. Therefore, histopathology and immunohistochemistry (IHC) for the FIPV antigen is still the gold standard for FIP diagnosis. In ocular cases specifically, the etiologic diagnosis solely based on histopathology of enucleated globes is significantly challenging, and a false positive diagnosis carries serious implications due to the likelihood of euthanasia [1,16]. Though advanced testing modalities are available, reported success of IHC labelling for the viral antigen in cases of FIP ophthalmitis has been limited [18,24], and detection of viral RNA by RT-qPCR or in situ hybridization (ISH) in ocular tissues has not been previously reported. We hypothesized that the odds of diagnosing FIP-associated ophthalmitis via RT-qPCR, antigen detection by IHC or viral RNA detection by ISH is significantly correlated with the inflammatory pattern. Here, we describe a case series of FIP-associated ophthalmitis (n = 30) in which we compared RT-qPCR, IHC and RNAscope® ISH for the detection of FIPV in the uveal tract and its correlation with the type of inflammatory pattern within affected eyes. Our results demonstrate that the later stages of the disease, which are characterized by heavy plasmacytic inflammation, are associated with lower detection rates of the viral antigen and RNA, and therefore, the odds of confirming the diagnosis of FIP-induced uveitis are significantly lower when a plasma cell-rich infiltration predominates in affected eyes.

## 2. Results

### 2.1. Pathotype, Extraocular Lesions and Duration of Clinical Signs in Selected Cases

Pathotype, duration of clinical signs and extraocular findings are summarized in Table 1. Complete necropsy findings were available in 27 of 30 cases. A total of 22 cats had the dry form (81.4%; 22/27). Nephritis (74.0%; 20/27), meningoencephalitis (51.8%; 14/27), hepatitis (44.4%; 12/27), peritonitis (37.0%; 10/27) and pneumonia (25.9%; 7/27) were the most common non-ophthalmological lesions identified in these cats. Other lesions included lymphadenitis (22.2%; 6/27), hyperplasia of secondary lymphoid tissues (18.5%; 5/27), pleuritis (14.8%; 4/27), lymphoid depletion (7.4%; 2/27), pericarditis (7.4%; 2/27), pancreatitis (3.7%; 1/27) and carpal osteomyelitis (3.7%; 1/27). Of the 14 cases with meningoencephalitis, 11, 2 and 1 had the dry, mixed and wet forms, respectively (Table 1).

The duration from onset of clinical signs to euthanasia or death was reported in 26 cases, ranging from 1 day to 24 weeks, with a median of 3 weeks (interquartile range = 3 weeks; Table 1). Nine cases had greater than 3 weeks duration; six of these had the dry form, two had the mixed form, and one had the wet form.

### 2.2. Ocular Lesions in Cats with FIP-induced Uveitis

**Figure 1:**
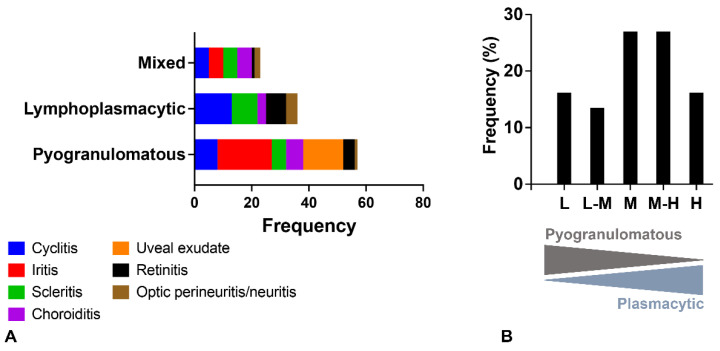
(**A**) Predominance of ocular inflammatory lesions by nature of the inflammatory infiltrate and anatomical location; (**B**) frequency (%) based on plasma cell abundance ([low; L] ≥70% pyogranulomatous and <30% plasmacytic; [low to medium; L-M] 50–70% pyogranulomatous and 30–50% plasmacytic; [medium; M] equally mixed pyogranulomatous and plasmacytic; [medium to high; M-H] 30–50% pyogranulomatous and 50–70% plasmacytic; [high; H] <30% pyogranulomatous and ≥70% plasmacytic).

**Figure 2:**
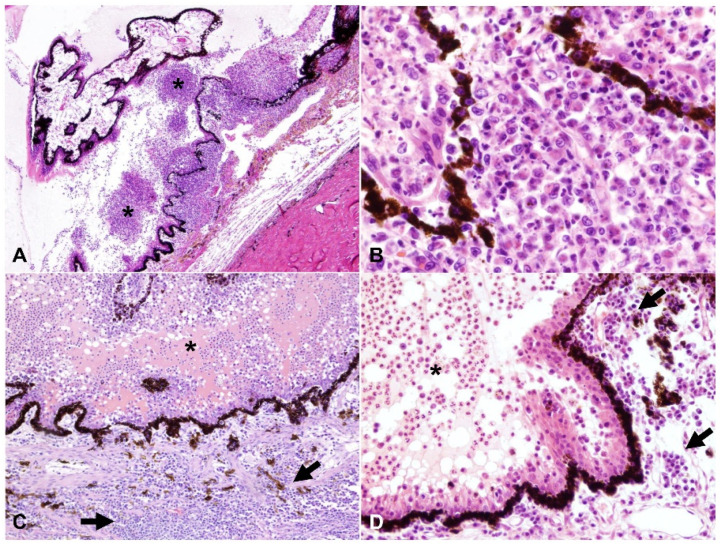
Cyclitis with variable pyogranulomatous and plasmacytic inflammation, H and E: (**A–D**) the posterior chamber is filled with intense pyogranulomatous exudate (asterisks), while the stroma of the pars plicata of the ciliary body is markedly expanded by either similar pyogranulomatous (**A**,**B**) or by plasmacytic infiltrate (arrows, (**C**) and (**D**)); (**A**) 40× total magnification; (**B**) 200× total magnification; (**C**) 100× total magnification; (**D**) 200× total magnification.

**Figure 3:**
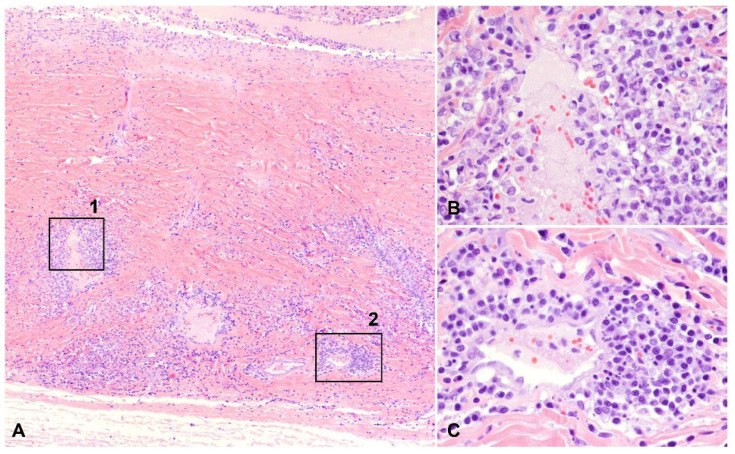
Pyogranulomatous and plasmacytic scleral vasculitis, H and E: (**A**) inflammatory cells infiltrate the vascular wall and the surrounding stroma in the sclera (40× total magnification); (**B**) mural and perivascular pyogranulomatous infiltration, magnified from squared area 1 noted on (**A**) (400× total magnification); (**C**) plasmacytic infiltration centered on a blood vessel, magnified from squared area 2 noted on (**A**) (400× total magnification).

**Figure 4:**
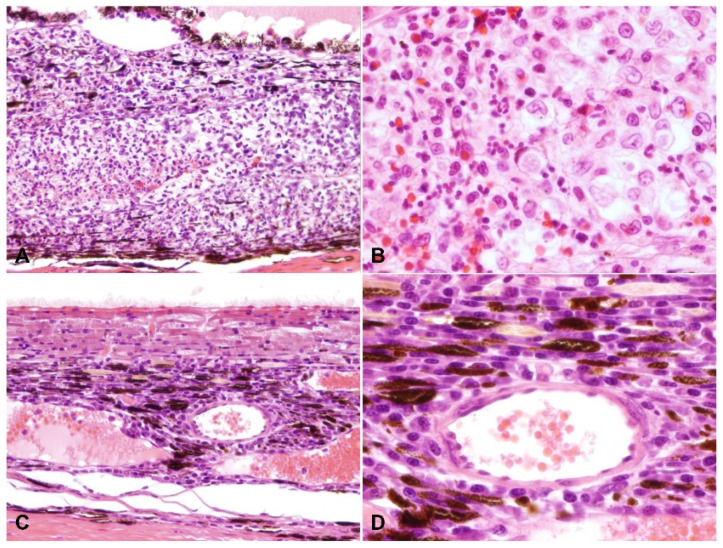
Choroiditis. H and E: (**A**) the choroid is markedly expanded by pyogranulomatous inflammation (200× total magnification); (**B**) macrophages and neutrophils infiltrate the choroid (400× total magnification); (**C**) the choroid is expanded by plasmacytic infiltrate (200× total magnification); (**D**) higher magnification of (**C**) showing perivascular infiltration of plasma cells (400× total magnification).

**Figure 5:**
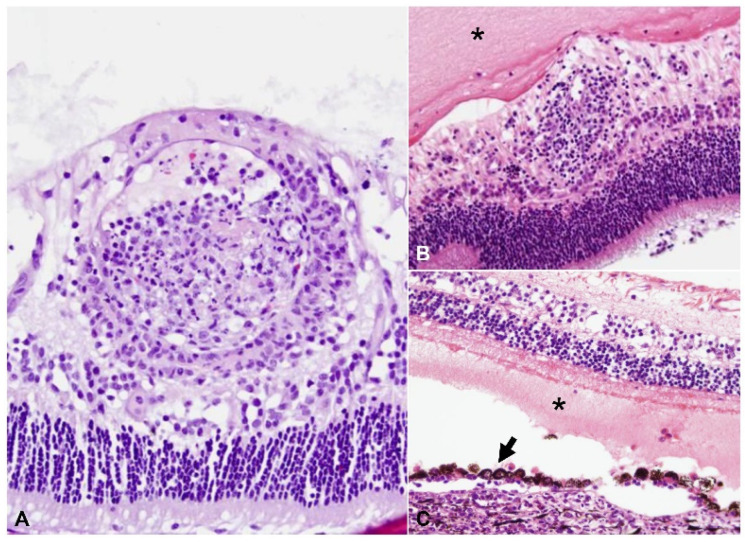
Retinitis. H and E: (**A**) pyogranulomatous vasculitis, with thrombosis, perivascular edema, fibrinous exudation and vascular and perivascular infiltration of macrophages, plasma cells and neutrophils (200× total magnification); (**B**) there is perivascular lymphoplasmacytic infiltrate; note the marked proteinaceous exudate in the posterior segment (asterisk, 200× total magnification); (**C**) there is retinal detachment with subretinal exudate (asterisk) and hypertrophy (“tomb stoning”) of the retinal pigment epithelium (arrow) (200× total magnification).

**Figure 6:**
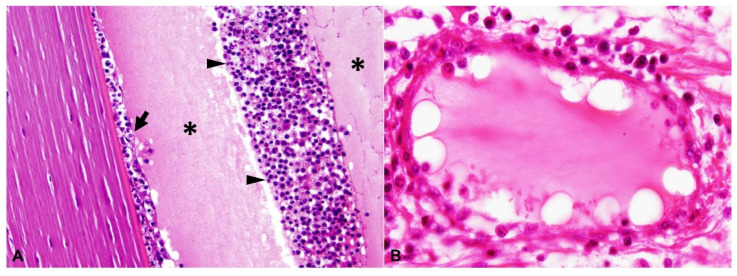
(**A**) Keratic precipitates (arrow) and hypopyon (arrowheads) with accompanying proteinaceous exudate (asterisks) in the anterior chamber; H and E, 400× total magnification; (**B**) Plasmacytic vasculitis as characterized by plasmacytic infiltrate into the vessel wall with fibrinoid mural degeneration and perivascular fibrinous exudate; H and E, 400× total magnification.

A summary of the histologic lesions is presented in Figure 1. The most common site of ophthalmitis was the anterior uvea, with cyclitis (Figure 2) in 86.7% (26/30) of cases and iritis in 80.0% (24/30). Twenty percent (6/30) of cases had a pre-iridal fibrovascular membrane. Scleritis (Figure 3) was present in 63.3% (19/30) of cases, while 16.7% (5/30) had keratitis. The posterior uvea (Figure 4) was the second most commonly involved intraocular site (46.7%; 14/30), followed by the retina (Figure 5) (40.0%; 12/30). Proteinaceous or fibrinous exudate was present in the anterior or posterior segment of 63.3% (19/30) of globes (Figure 5 and Figure 6); keratic precipitates and hypopyon (Figure 6) were present in 46.7% (14/30) and 26.7% (8/30) of cases, respectively. The optic nerve was included in the sections of 13 globes from 12 cases. Within these, optic neuritis was present in 1/12 cases (8.3%), and inflammation of the optic nerve-associated meninges was present in 7/12 cases (58.3%).

Frequency of pyogranulomatous vs. plasma cell-rich inflammation in the uvea is shown in Figure 1. Inflammation within the anterior uvea was predominantly plasmacytic in all 24 cases with iritis and in 88.5% (23/26) of cases with cyclitis. The infiltrating inflammatory cells in the iris mostly expanded the posterior aspect of the iris with less infiltration of the anterior stroma. The plasmacytic infiltrate within the ciliary body was often more heavily concentrated in the pars plicata (Figure 2), and although there was commonly peri-uveal exudate overlying the ciliary body (14/30 cases overall, and 14/26 cases with cyclitis), the plasma cells remained within the uveal stroma or epithelium in each case. The exudate overlying the pars plicata of the ciliary body and posterior iris epithelium was pyogranulomatous in all 14 cases in which it was noted (Figure 2). In most cases, this peri-uveal exudate was concentrated in the anterior peripheral vitreous and present to a lesser extent in the posterior chamber. The inflammatory infiltrate within the uveal tract displayed a superior–inferior polarity in 17 cases, with more severe inflammatory infiltrate in the superior (tapetal) uveal tract in 12 of these 17 cases. In the eight cases in which both eyes were available for examination, the degree of inflammation was similar between the two.

A total of 9 of the 30 cases (30.0%) had vasculitis, 8 of which consisted of pyogranulomatous infiltration of the vessel wall (Figure 3 and Figure 5), with the remaining cases characterized by plasmacytic infiltrate (Figure 6). Regardless of the infiltrate, all lesions of vasculitis included fibrinoid degeneration of the vessel walls, with plump activated and/or necrotic endothelial cells. Vasculitis was noted in the episcleral vessels in five cases: in the choroid, ciliary body, and retina in four cases each and in the iris in two cases.

In the 14 cases with neurologic disease in our series, anterior uveitis (cyclitis (13/14), iritis (11/14)), exudate in the anterior chamber (11/14), peri-uveal exudate (9/14), scleritis (9/14) and keratic precipitates (8/14) were common, while retinitis was relatively uncommon (5/14).

### 2.3. Detection of Viral Antigen and RNA Via Immunohistochemistry, RNAscope® In Situ Hybridization and RT-qPCR

**Figure 7:**
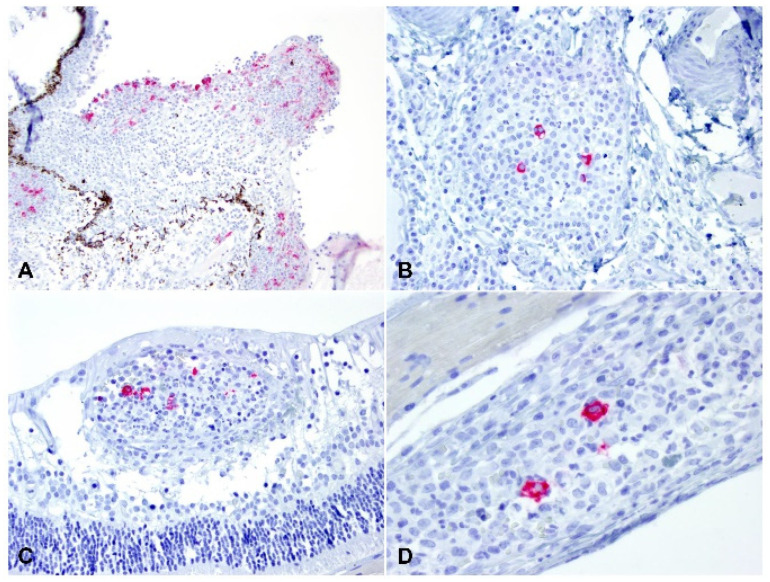
Immunohistochemical labelling for the feline coronavirus antigen, AEC (red): (**A**) the viral antigen is detected in the inflammatory cells within the ciliary body and peri-uveal exudate (100× total magnification); (**B**) the meninges lining the optic nerve are infiltrated by macrophages containing the viral antigen (200× total magnification); (**C**) macrophages containing the viral antigen are present in the retinal vessels; and (**D**) in the choroid (100× and 200× total magnification).

**Figure 8:**
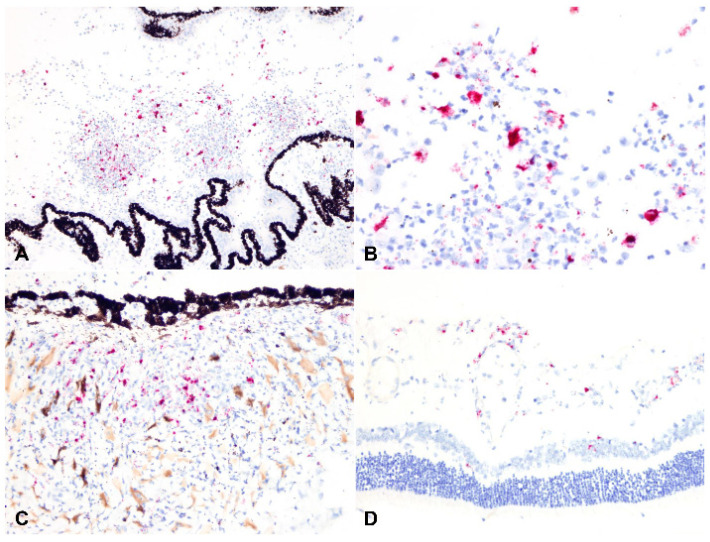
RNAscope® in situ hybridization for feline coronavirus RNA, Fast Red (red): (**A**) peri-uveal exudate with macrophages containing viral RNA (100× total magnification); (**B**) higher magnification of (**A**), the viral RNA labeling is cytosolic in macrophages (400× total magnification). Macrophages containing viral RNA are present in the iris (**C**) and retina (**D**), 200× total magnification.

**Figure 9:**
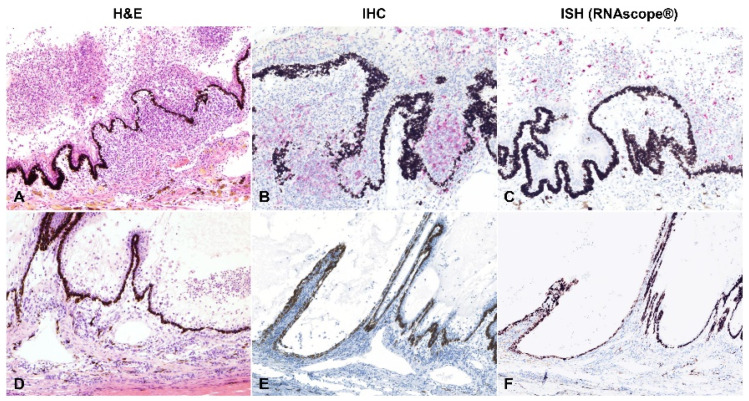
Correlation between the type of inflammatory infiltration and the viral antigen or viral RNA detection. The case in the upper row (**A**–**C**; case 13a) has predominantly pyogranulomatous ophthalmitis (**A**); ≥70% pyogranulomatous and <30% plasmacytic). Abundant viral antigen (**B**) and viral RNA (**C**) were detected by immunohistochemistry (IHC) and in situ hybridization (ISH), respectively. The case in the bottom row (D-F; case 23a) has predominantly plasmacytic ophthalmitis (**D**; 30–50% pyogranulomatous and 50–70% plasmacytic). No viral antigen (**E**) or viral RNA (**F**) were detected by IHC or ISH, respectively. AEC (IHC) and Fast Red (ISH); (**A**,**D**–**F**) 100× total magnification; (**B**,**C**) 200× total magnification.

The viral antigen and RNA were detected via IHC and ISH, respectively. Overall, 30.0% (9/30) cases had detectable viral antigen, and 33.3% (10/30) had detectable viral RNA. A total of 8/9 and 9/10 of these cases were classified as the dry form, with only a single case representing the wet form. The viral antigen and RNA were noted bilaterally in three of the five cases in which both eyes were assessed by IHC and ISH (see below Section 4). The positive labelling was cytoplasmic in macrophages within pyogranulomatous inflammation (Figure 7, Figure 8 and Figure 9). The macrophages containing the coronavirus antigen and viral RNA were in the anterior uvea, choroid, peri-uveal exudate, pre-iridal fibrovascular membrane and retina. Five cases with demonstrable viral antigen and viral RNA had vasculitis. Vasculitis in cases with positive IHC and ISH was present in the anterior uvea, choroid and retina. The majority of the eyes in which the viral antigen and RNA were detected had mostly moderate to severe (>50%) pyogranulomatous inflammation.

Approximately one-fourth (26.7% or 8/30) of the evaluated cases yielded a positive result by RT-qPCR. Four of these cases were among those in which both eyes were examined, and in all four of these cases, FIPV RNA was identified in tissue from both eyes. Among the RT-qPCR-positive cases, seven of eight were positive by IHC, and all (eight of eight) were positive by ISH. Testing results for the 30 cases are summarized in Table 2, Table 3, Table 4 and Table 5. The overall agreement (kappa statistic) was high between RT-qPCR and IHC (Table 3), RT-qPCR and ISH (Table 4), and IHC and ISH (Table 5; 0.754 [CI95% 0.492–1], 0.842 [CI95% 0.633–1], and 0.769 [CI95% 0.523–1], respectively). Correlation analysis determined a strong positive correlation between tests (ρ = 0.756, *p*-value < 0.001 between RT-qPCR and IHC; ρ = 0.808, *p*-value < 0.001 between IHC and ISH as well as between RT-qPCR and ISH).

### 2.4. Relationship between Type of Inflammation and Detection of Viral Antigen and RNA via Immunohistochemistry, RNAscope® In Situ Hybridization and RT-qPCR

Based on the hypothesis that the odds of diagnosing FIP-associated ophthalmitis via RT-PCR, antigen detection by IHC or viral RNA detection by ISH is significantly correlated with the inflammatory pattern, we evaluated the association between the type of inflammation, its severity, and testing results (RT-qPCR, IHC and ISH). A rank biserial correlation analysis identified a weak to moderate negative correlation between type of inflammation (as described under Materials and Methods) and RT-qPCR (r_rb_ = −0.380, *p*-value = 0.02), IHC (r_rb_ = −0.466, *p*-value = 0.004) and ISH results (r_rb_ = −0.373, *p*-value = 0.028), respectively. Therefore, this is indicative of a significant relationship between these factors and suggests that as the plasmacytic response becomes predominant within the eyes of infected cats, the likelihood of obtaining a positive and confirmatory test result by RT-qPCR, IHC or ISH decreases (Figure 9). Interestingly, the severity of the inflammation was negatively correlated with the inflammation type (r_rb_ = −0.354, *p*-value = 0.032; i.e., the pyogranulomatous response is often of more intensity than the plasmacytic-type) and positively correlated with positive testing results by RT-qPCR, IHC or ISH (r_rb_ = 0.407, 0.554 and 0.464 for RT-qPCR, IHC and ISH, respectively, [*p*-values = 0.012 to < 0.001]; i.e., the odds of confirming FIP by additional laboratory testing are higher with increased severity in the inflammatory response).

## 3. Discussion

FIP continues to be a significant health burden in feline medicine, with significant challenges in diagnosis, prevention and treatment [4,23]. FIPV infection has a complex pathogenesis that is not fully understood, and systemic disease develops due to the altered tropism of this coronavirus, which infects the monocyte—macrophage cell lineage [2,3,7,8,9,10,25,26,27,28,29,30,31]. Lesions associated with FIP are, to a certain extent, correlated with the type of immune response developed by the host. It is well-known that both central nervous system and ocular lesions tend to occur most frequently with the dry form of FIP [13,19,32]. Antemortem confirmatory diagnosis imparts significant challenges for the feline practitioner, as there is no single accurate test for diagnosis, and available tests (e.g., RT-qPCR) often lack sufficient sensitivity and specificity or may be influenced by sample type [21,22]. In addition, the time point within the course of the disease and consequently, the predominant inflammatory response at the time of testing could also be factors associated with the success or failure in confirmatory testing of FIP. Therefore, we hypothesized that the type of inflammatory response (i.e., predominantly pyogranulomatous vs. predominantly plasmacytic) influences the chances of confirming the diagnosis of FIP by IHC, ISH or RT-qPCR. Here, we describe the ocular findings in 30 cases of FIP-associated ophthalmitis and attempt to determine the relationship between the inflammatory cell response and the likelihood of detecting the FIP antigen and RNA within the affected eyes.

A recent study has provided high-level detail of the ocular lesions in cats affected by FIP, demonstrating that nearly all components of the three ocular tunics can be affected during this systemic disease [18]. That study determined that, while the inflammatory response is variable, B lymphocytes/plasma cells are often more abundant than macrophages within ocular inflammatory infiltrates and that the latter are often increased when vascular damage is present [18]. Our histological findings correlate with the observations in this recent study. Approximately one-third of the cases evaluated here had intense pyogranulomatous inflammation, while the remaining globes had either mixed pyogranulomatous/plasmacytic or predominantly plasmacytic inflammation. Previously described histopathologic lesions include protein-rich, cell-poor exudate in the anterior and posterior segments [24]; pyogranulomatous anterior uveitis ± chorioretinitis, occasional optic meningitis or neuritis [24,32] and keratitis [32]. In contrast to previous reports [32], we found that choroiditis (46.7%) and retinitis (40.0%) did not occur in the majority of specimens. Whether these differences are strictly due to the planes of section examined or are the result of different patient characteristics or viral strains is unknown.

A previous study of 16 cats with the neurologic form of FIP described 5 cats with ocular lesions characterized as anterior uveitis (5/5 cats), keratic precipitates (3/5) and retinitis (1/5) [33]. The 14 cases with neurologic disease in our series presented similar findings, with the addition of intraocular exudate and scleritis as common findings, though these findings were also common in the other 16 cases included in this study.

Amongst all 30 cases, we found the following unique characteristics in cases of ophthalmitis attributed to FIP: (1) cases with moderate to marked cyclitis often had interstitial and intraepithelial plasmacytic infiltrate, with pyogranulomatous peri-uveal exudate overlying the pars plicata (Figure 2); (2) there was more severe inflammatory infiltrate in the superior uveal tract in 12/17 cases that displayed superior–inferior polarity; and (3) the incidence of scleritis in the cases examined was relatively high (19/30 cases, 63.3%).

IHC has been reported to be a diagnostic technique with high accuracy [34]; thus, histopathology and immunohistochemistry are considered the gold standard in the confirmatory diagnosis of FIP. IHC has been reportedly unreliable in cases of FIP ophthalmitis, however [24]. In our study, the viral antigen was detected in only 9/30 cases, with very similar detection rates and high agreement with RT-qPCR and ISH. Thus, we hypothesized that the predominant type of inflammatory response can determine the likelihood of the viral antigen (and RNA) detection in cases of FIP ophthalmitis. Our study demonstrated that while weak to moderate, there is a significant negative association between the abundance of plasma cell-rich inflammation in the uvea of affected cats and the low detection rate of the viral antigen and RNA via IHC, ISH and RT-qPCR in ocular tissues. Even though the association is not strong, this observation carries diagnostic relevance for the pathologist and informs testing decisions in cases in which there is intense uveal plasmacytic response and, consequently, lower chances of confirmatory diagnosis by these techniques. Nevertheless, loss of antigenicity and RNA degradation associated with long archiving and/or fixation in Bouin’s fixative cannot be ruled out and could have had an impact in the rate of detection in this study [35]. This factor in addition to the limited number of globes examined (30 cases) could have had an impact in the only weak to moderate strength of this association.

An additional novel aspect of this study is the use of RNAscope® ISH for the detection of FIP in ocular tissues. To date, this method had not been described in the literature. Here, while in high agreement with IHC and RT-qPCR, it has proven to have the highest detection rate among the three diagnostic techniques used (10/30). The sensitivity and specificity of this technology has been well described and validated for other pathogens [36,37,38,39,40,41,42,43,44,45] and therefore could be a promising tool for the diagnosis of FIP in tissue sections. Further evaluation of this method in extraocular tissues from FIP-infected cats is warranted to determine its performance.

In conclusion, this study describes ocular findings in 30 FIP cats and is the first to establish a negative association between the abundance of the plasmacytic inflammatory response and the success rate in detection of FIP virus antigen and RNA. FIP should be suspected based on ocular histopathologic findings of proteinaceous exudate, episcleral vasculitis or perivascular inflammation, anterior uveitis, pyogranulomatous peri-uveal exudate and a preferential distribution of the inflammation in the superior (tapetal) uveal tract. Additional findings include pyogranulomatous vasculitis of the sclera, uvea and retina. This study further suggests that the likelihood of a confirmatory diagnosis of FIP by IHC, ISH or RT-qPCR in ocular tissues is low in cases in which there is intense plasmacytic ophthalmitis. This is of significance to veterinary pathologists and feline practitioners at the time of interpreting ancillary tests results, which need to be cautiously interpreted with consideration of the histologic alterations. Based on the high level of agreement between RT-qPCR, IHC and ISH, it is likely that these assays have similar diagnostic performance for FIP. Additional comparative performance evaluation of these assays is warranted.

## 4. Materials and Methods

### 4.1. Case Selection and Histopathology

Formalin-fixed paraffin-embedded (FFPE) sections of one eye obtained from 22 domestic cats, and both eyes from 8 domestic cats were included in this study. Selection criteria included gross, histologic and immunohistochemical diagnosis of FIP, and significant histologic inflammatory ocular lesions in the absence of evidence of another cause for the ocular lesions. Necropsy reports [Cornell University, College of Veterinary Medicine, Dept. of Biomedical Sciences, Ithaca, NY (n = 22) and Michigan State University Veterinary Diagnostic Laboratory, College of Veterinary Medicine (n = 5)] were reviewed for duration of clinical signs, extraocular lesion distribution and pathotype identified during necropsy. The globes had been fixed in 10% neutral buffered formalin, Zenker’s solution or Bouin’s solution and were routinely paraffin embedded. Five-micrometer thick sections were stained with hematoxylin and eosin. The lesions were characterized based on anatomic location of inflammation and the type of infiltrating cells. The inflammatory infiltrate was semiquantitatively categorized based on the abundance of plasma cell infiltrate in the uveal tract as follows: (low) ≥70% pyogranulomatous and <30% plasmacytic; (low to medium) 50–70% pyogranulomatous and 30–50% plasmacytic; (medium) equally mixed pyogranulomatous and plasmacytic; (medium to high) 30–50% pyogranulomatous and 50–70% plasmacytic; (high) <30% pyogranulomatous and ≥70% plasmacytic. This distinction was drawn based on published studies indicating that plasma cells infiltrate pyogranulomas of FIP and eventually become the predominant cell type [46]. Overall lesion severity was semiquantitatively scored from 1 (mild) to 3 (severe).

### 4.2. Immunohistochemistry (IHC)

IHC was performed in all eyes included in the study (single eyes from 22 cats and both eyes from 8 cats). For indirect immunohistochemical evaluation, 5 μm thick sections of the formalin-fixed, paraffin-embedded tissues used for histopathology were mounted on negatively charged glass slides. The immunostainer used was a DAKO automatic universal staining system. Slides with tissue sections were heated in a 60 °C oven for 1 h to help adherence of the paraffin-embedded tissue sections to the slides. Depararaffinization and rehydration of slides were obtained in several changes of xylenes and progressive decreasing grades of ethanol. Heat-mediated antigen retrieval, in buffer pH 9.0, for 15 min, in a 200 W microwave oven was performed before staining. Hydrogen peroxide was applied to tissue sections for 10 min to inactivate endogenous peroxidases. An FIP anti-nucleocapsid-specific mouse monoclonal antibody (clone FIPV3-70 [47]) was diluted 1:10 and applied to sections for 30 min at room temperature. For the immunodetection, the LSAB2 kit from DAKO was used. The kit utilizes a secondary goat anti-mouse biotinylated immunoglobulin for 15 min, followed by a streptavidin–horseradish peroxidase (HRP) conjugate that is applied for 15 min. Visualization of antibody binding was obtained via 3-amino-9-ethylcarbazole (AEC) substrate chromogen for 5 min. Following AEC incubation, sections were rinsed with deionized water, counterstained manually with Mayer’s hematoxylin (3 min), coverslipped with permanent mounting medium and examined with a light microscope. Positive control tissues from a cat with confirmed FIP based upon histopathology, a direct fluorescent antibody test and an indirect immunohistochemistry was included in each run to confirm immunoreactivity of the appropriate pattern. Negative isotype antibody control was included with each test slide to ensure the lack of nonspecific binding. IHC for FIPV antigen was performed in multiple organs in each case besides the eyes in order to confirm the diagnosis of FIP.

### 4.3. RNAscope® In Situ Hybridization (RNAscope® ISH)

RNAscope® ISH was performed in all single eyes from 22 cats and both eyes from 5/8 cats, while for the remaining 3 cats only a single eye was available for this test procedure due to sample shortages. For FIPV RNAscope® ISH, an anti-sense probe targeting ORF1ab (nucleotide sequence: 12,380–13,396 of FIPV isolate FCoVWSU791146_P1 (GenBank accession number KC461235.1) was purchased from Advanced Cell Diagnostics (Newark, CA, USA). The RNAscope® ISH assay was performed as previously described [48] using the RNAscope 2.5 LSx Reagent Kit (ACD, Newark, CA, USA) on the automated BOND RXm platform (Leica Biosystems, Buffalo Grove, IL, USA). Briefly, five-micron sections of formalin-fixed paraffin-embedded (FFPE) tissue were subjected to automated baking and deparaffinization followed by heat-induced epitope retrieval (HIER) using a ready-to-use EDTA-based solution (pH 9.0; Leica Biosystems, Buffalo Grove, IL, USA) at 100 °C for 15 min. Subsequently, tissue sections were treated with a ready-to-use protease (RNAscope® 2.5 LSx Protease [ACD, Newark, CA, USA]) for 15 min at 40 °C, followed by a ready-to-use hydrogen peroxide solution for 10 min at room temperature. Slides were then incubated with the ready-to-use probe mixture for 2 h at 40 °C, and the signal amplified using a specific set of amplifiers (AMP1 through AMP6 as recommended by the manufacturer). The signal was detected using a Fast-Red solution for 10 min at room temperature. Slides were counterstained with ready-to-use hematoxylin for 5 min, followed by five washes with 1X BOND Wash Solution (Leica Biosystems) for bluing. Slides were finally rinsed in deionized water, dried in a 60 °C oven for 30 min, and mounted with Ecomount® (Biocare, Concord, CA, USA). Sections from the lung of an infected cat confirmed by histopathology, immunofluorescence and IHC were used as a positive assay control. A negative control probe targeting dihydrodipicolinate reductase (DapB) was used to assess non-specific binding.

### 4.4. RT-qPCR

RT-qPCR was performed on all eyes included in the study (single eyes from 22 cats and both eyes from 8 cats). Total RNA from FFPE tissue sections was purified using the RNeasy FFPE Kit (Qiagen Inc., Valencia, CA, USA), following the manufacturer’s protocol. Extracted RNA samples were tested for the presence of feline coronavirus RNA by using a real-time (RT-qPCR assay developed and used routinely at the Michigan State University Veterinary Diagnostic Laboratory, East Lansing, MI, USA). The forward primer (5′-ACGTGTAATRGGAGGTACAAG-3′), reverse primer (5′-TAGCTCTTCCATTGTTGGCTC-3′) and the probe (FAM5’-CTTCTCTAAATTACTAAATCTAGCATTGCCAA-3’BHQ-1) target the 3’ untranslated region (UTR) of the genome that is conserved among feline and canine coronaviruses and transmissible gastroenteritis virus of swine. The amplicon size is 129 bp. The AgPath-ID One-step RT-PCR Kit (Life Technologies, Foster City, CA, USA) was used according to the manufacturer’s instructions, with primers at 0.5 µM and probe at 120 nM final concentrations in a 25 µL reaction volume. Real-time amplification was performed with the Applied Biosystems 7500 Fast real-time PCR system (Life Technologies, Carlsbad, CA, USA) in standard mode with the following cycling parameters: reverse transcription at 45 °C for 10 min, pre-denaturation at 95 °C for 10 min and 45 cycles of 94 °C for 10 s, 53 °C for 30 s and 72 °C for 10 s, with optical data collection at the 53 °C annealing step.

### 4.5. Data Analysis

A rank biserial correlation analysis was performed using SPSS Statistics (SPSS Inc., Chicago, IL, USA) to determine whether there was a significant association between the type of inflammatory response and the IHC, ISH and RT-qPCR results. P-values <0.05 were considered significant. Agreement between diagnostic assays was determined based on the kappa statistic and calculated using GraphPad QuickCalcs tool.

## Figures and Tables

**Table 1:** Pathotype, duration of clinical signs and extraocular findings in cats with FIP-associated uveitis (n = 30).

Animal Identification	Pathotype	Duration (Weeks)	Extraocular Findings
1	Wet	4	Fibrinous peritonitis
2a	Dry	1	Pyogranulomatous and fibrinous meningoencephalitis
2b			
3	Wet	1	Granulomatous peritonitis; lymphoplasmacytic pancreatitis
4	Wet	2	Necrotizing and plasmacytic pleuritis and peritonitis; fibrinosuppurative and plasmacytic hepatitis; necrotizing and plasmacytic interstitial nephritis; pyogranulomatous pancreatitis
5a	Wet	3	Nonsuppurative epicarditis; granulomatous meningitis; fibrinous and pyogranulomatous pleuritis and peritonitis; granulomatous nephritis
5b			
6	Dry	1	Pyogranulomatous nephritis, hepatitis, mesenteric lymphadenitis, and meningitis; nonsuppurative interstitial pneumonia
7	Dry	7	Granulomatous peritonitis, meningitis, polyradiculoneuritis, and nephritis
8	Dry	24	Suppurative lymphadenitis
9	Dry	3	Pyogranulomatous nephritis, hepatitis, meningitis
10	Dry	3	Chronic cholangitis; fibrinous splenitis; peripancreatic necrosis; eosinophilic and granulomatous pancreatic lymphadenitis; eosinophilic osteomyelitis (carpi)
11	Wet	NA	Chronic interstitial pneumonia; fibrinous, pyogranulomatous peritonitis; pyogranulomatous cholangiohepatitis
12	Mixed	NA	Nonsuppurative meningoencephalitis; necrotizing adrenalitis; nonsuppurative renal vasculitis; mesenteric lymphadenitis
13a	Dry	0.14	Granulomatous and necrotizing peritonitis, nephritis, splenitis, hepatitis, pneumonia, enterocolitis, lymphadenitis, pituitary adenitis, and encephalitis
13b			
14a	Dry	6	Granulomatous nephritis, hepatitis, encephalomyelitis; fibrinous peritonitis
14b			
15a	Dry	1	Granulomatous nephritis, hepatitis, encephalitis
15b			
16	Dry	3.5	Pyogranulomatous nephritis, hepatitis, peritonitis, pleuritis, pericarditis, and pneumonia
17	Dry	4	Pyogranulomatous pleurtitis, epicarditis, pulmonary vasculitis, meningitis, neuritis, nephritis, and splenitis
18	Wet	1	Granulomatous peritonitis, nephritis and mesenteric lymphadenitis
19	Dry	1	Nonsuppurative meningoencephalitis and perivascular nephritis; purulent rhinitis
20	Dry	3	Lymphocytic nephritis and periportal hepatitis
21	Dry	5	Granulomatous and necrotizing meningoencephalomyelitis; pyogranulomatous nephritis
22	Dry	2	Granulomatous nephritis; lymphoma
23a	Mixed	6	Pyogranulomatous meningoencephalitis; subacute purulent pneumonia; pyogranulomatous nephritis
23b			
24	Dry	0.28	Pyogranulomatous serositis and nephritis, bronchointerstitial pneumonia, hepatitis
25	Dry	NA	NA
26a	Dry	NA	NA
26b			
27	Dry	16	NA
28	Dry	3	Pyogranulomatous nephritis and hepatitis
29	Dry	NA	Meningoencephalitis
30a	Dry	0.7	Pyogranulomatous and plasmacytic meningitis, pneumonia, peritonitis, nephritis
30b			

NA, not available; a and b refer to opposite eyes from the same animal in those cases in which both globes were available.

**Table 2:** Grading of uveal inflammation by cellular composition and severity, and RT-qPCR, IHC and ISH results for FIP in the eyes of 30 cats with FIP-associated uveitis.

Animal Identification	Cellular Composition	Grade–Severity	RT-qPCR	IHC	ISH
1	H	1	-	-	-
2a	M	3	+	+	+
2b	M	3	+	+	+
3	L	2	+	+	+
4	L	1	-	-	-
5a	M-H	2	-	-	-
5b	M	3	-	-	NA
6	L-M	3	-	-	-
7	M-H	2	-	-	+
8	H	1	-	-	-
9	H	1	-	-	-
10	L	2	-	-	-
11	L	2	-	-	-
12	M	1	-	-	-
13a	L-M	2	+	+	+
13b	L-M	1	+	-	-
14a	M	3	+	+	+
14b	M	3	+	+	NA
15a	M-H	2	-	-	-
15b	H	2	-	-	-
16	M	3	-	+	-
17	L	2	-	+	+
18	M-H	2	-	-	-
19	H	1	-	-	-
20	M-H	3	-	-	-
21	M-H	1	-	-	-
22	L-M	3	-	-	-
23a	M-H	2	-	-	-
23b	H	1	-	-	-
24	L-M	3	+	+	+
25	L	3	+	+	+
26a	M-H	2	-	-	-
26b	NA	NA	-	-	NA
27	M-H	3	-	-	-
28	M	2	-	-	-
29	M-H	2	+	-	+
30a	M	3	+	+	+
30b	M	3	+	+	+

a and b indicate separate eyes from the same animal; NA, not available.The inflammatory infiltrate was semiquantitatively categorized based on the degree of pyogranulomatous and plasmacytic inflammation of the uveal tract as a whole as follows: (low; L) ≥70% pyogranulomatous and <30% plasmacytic; (low to medium; L-M) 50–70% pyogranulomatous and 30–50% plasmacytic; (medium; M) equally mixed pyogranulomatous and plasmacytic; (medium to high; M-H) 30–50% pyogranulomatous and 50–70% plasmacytic; (high; H) <30% pyogranulomatous and ≥70% plasmacytic. Lesion severity was semiquantitatively scored from 1 (mild) to 3 (severe).

**Table 3:** Contingency table analysis between different methods for FIP detection utilized in the eyes of infected cats with FIP-induced uveitis (n = 30): comparison between RT-qPCR and IHC; Kappa statistic and 95% confidence interval [CI 95%]) are indicated below.

		IHC		
		Positive	Negative	Total
**RT-qPCR**	**Positive**	7	1	8
	**Negative**	2	20	22
	**Total**	9	21	30

Kappa = 0.754; CI 95%: 0.492–1.

**Table 4:** Contingency table analysis between different methods for FIP detection utilized in the eyes of infected cats with FIP-induced uveitis (n = 30): comparison between RT-qPCR and ISH. Kappa statistic and 95% confidence interval [CI 95%]) are indicated below.

		ISH		
		Positive	Negative	Total
**RT-qPCR**	**Positive**	8	0	8
	**Negative**	2	20	22
	**Total**	10	20	30

Kappa = 0.842; CI 95%: 0.633–1.

**Table 5:** Contingency table analysis between different methods for FIP detection utilized in the eyes of infected cats with FIP-induced uveitis (n = 30): comparison between IHC and ISH. Kappa statistic and 95% confidence interval [CI 95%]) are indicated below.

		ISH		
		Positive	Negative	Total
**IHC**	**Positive**	8	1	9
	**Negative**	2	19	21
	**Total**	10	20	30

Kappa = 0.769; CI 95%: 0.523–1.

## Data Availability

Not applicable.
